# Swiping right: face perception in the age of Tinder

**DOI:** 10.1016/j.heliyon.2019.e02949

**Published:** 2019-12-02

**Authors:** Antonio Olivera-La Rosa, Olber Eduardo Arango-Tobón, Gordon P.D. Ingram

**Affiliations:** a Department of Psychology and Social Sciences, Universidad Católica Luis Amigó. Transversal 514A #67B 90, Medellín, Colombia; b Human Evolution and Cognition Group, associated group to IFISC (University of the Balearic Islands – CSIC), Carr. de Valldemossa, km 7,5, Palma de Mallorca, 07122, Spain; c Department of Psychology, Universidad de Los Andes, Cra. 1 #18a-12, Bogotá, Colombia

**Keywords:** Tinder, Moral character, Face perception, Uncanny valley, Psychology

## Abstract

With an estimated 50 million or more users worldwide, Tinder has become one of the most popular mobile dating applications. Although judgments of physical attractiveness are assumed to drive the “swiping” decisions that lead to matches, we propose that there is an additional evaluative dimension driving behind these decisions: judgments of moral character. With the aim of adding empirical support for this proposition, we critically review the most striking findings about first impressions extracted from faces, moral character in person perception, creepiness, and the uncanny valley, as they apply to Tinder behavior. Drawing on this research and the evolutionary theory of biological markets, we formulate several hypotheses that offer directions for future studies of Tinder and other dating apps. We conclude that research on face perception of novel targets supports the plausibility of moral character as a potential factor affecting the swiping decisions and subsequent behavior of Tinder users.

## 1 Introduction

How people meet potential dating partners has changed due to the increasing usage of online dating. With an estimated 50 million users in more than 190 countries, 10 million daily active users and over 30 billion matches to date, Tinder has become one of the most popular mobile dating apps in the world (Tinder, 2019). Although it is promoted as a social discovery platform that “empowers users around the world to create new connections that otherwise might never have been possible” (Tinder, 2019), the view that Tinder is primarily a sex/hookup app which may also lead to romantic relationships remains prevalent among users and the general population (LeFabvre, 2018; Sumter et al., 2016; Orosz et al., 2018). Therefore, the fact that several different types of potential dating partners, looking for either short-term or long-term partnerships, might initiate contact through Tinder is relevant for investigating the psychological drivers underlying swiping decisions. In this article, we apply the evolutionary framework of biological markets to discuss one evaluative dimension that may play an important role in swiping decisions: judgments of moral character.

The goals of this article are: a) to discuss research on moral character and face perception in the context of behavior on Tinder; b) to propose moral character as an evaluative dimension driving swiping decisions; and c) to advance testable hypotheses that can guide future psychological research on Tinder. As a result, we intend to contribute to reaching a better understanding of the psychological mechanisms involved in decision-making on the Tinder platform and related online dating and social networking apps.

First, in order to contextualize our proposal, we briefly review some studies on the psychology of Tinder use and motives. Second, we introduce some relevant evolutionary psychological theories and explain why Tinder may be a particularly pure form of a biological market. Next, we discuss the most striking findings on the relationship between facial perception (which is a key feature of the Tinder format), moral character, and first impressions. We review relevant research that bears on the predicted role of moral character judgments in swiping decisions to see how this literature stands against a set of hypotheses related to Tinder behavior: H1) Social judgments extracted from faces should “colour” how non-face information about a person is processed; H2) Moral character judgments should be more likely to drive swiping decisions in female heterosexual users than in male heterosexual users; H3) Profile pictures that apply “unnatural” photo filters (e.g., big eyes) may facilitate social avoidance rather than social desirability; and H4) less immediately attractive mates need to do more “work” in the post-match phase in order to demonstrate their moral character and progress the match towards sexual contact.

It is worth mentioning, however, that this manuscript is intended as a research catalyst rather than a summative review (for a review on trait inferences from faces, see Olivola and Todorov, 2017), and hence these hypotheses remain open to further empirical testing.

## 2 A rapid look at Tinder: to swipe or not to swipe?

In order to better understand the factors that affect Tinder use, we start with a brief description of how Tinder works. Once downloaded, users have the possibility to synchronize their account with other social networks (e.g., Facebook, Instagram) or they can manually add some pictures and basic personal information (e.g., occupation, hobbies, educational background) to their Tinder profile. Although the basic version of Tinder is free, there are premium, subscription-based versions that enable new opportunities for the user (Tinder, 2019), and which could profoundly affect its use, perhaps turning it into something more like a traditional subscription-based dating site. In all cases, nonetheless, Tinder uses the location and the age of users as filters to offer them a particular dating pool, displayed one at a time as a sequence of profile photos associated with first names. As a result, “the user gets a photo and has to decide if he/she likes that person or not based on that photo^1^. If he/she likes that person, he/she has to swipe the picture of this person right. If he/she does not like that individual, he/she has o swipe left. If both parties like each other and swipe right, they are ‘matched’ and conversations begin” (Orosz et al., 2018, p.302).

In the last years, Tinder's increasing popularity has attracted the attention of psychologists. For instance, casual sex, love, friendship, self-esteem enhancement, ease of communication, boredom and trendiness have been identified as particular motivational factors behind Tinder use (Orosz et al., 2018; Sumter et al., 2016). Some results from an online survey suggest that, compared to women, men are more likely to use the app for casual sex and relationships (Sumter et al., 2016), and women rather for friendship and self-validation (Ranzini and Lutz, 2017). Women also appear to be more selective in their right-swiping decisions compared to men (Timmermans and Courtois, 2018). These results are in line with previous results showing that men are more likely to use social networks to form new relationships and find potential mates than women are (Muscanell and Guadagno, 2012; Mazman and Usluel, 2011; Raacke and Bonds-Raacke, 2008; see Section 3 for an evolutionary explanation of these findings).

Recently, it was found that unrestrictive sociosexuality (i.e., a preference for casual sex) is the major predictor of use of picture-based mobile dating apps, suggesting that Tinder may be merely a new scenario for enacting short-term mate-seeking behavior (Olav Botnen et al., 2018). In this vein, some studies showed that single Tinder users tend to be more extraverted and open to new experiences than single non-users (Timmermans and De Caluwé, 2017a, Timmermans and De Caluwé, 2017b); and that compared to non-users, Tinder users are risk-takers who have low sensitivity to sexual disgust (Sevi, 2018). Other research suggests that Tinder use could be associated with a variety of negative perceptions about body and self; for example, Tinder users may show lower levels of satisfaction with face and body and higher levels of appearance comparisons than non-users (Strubel and Petrie, 2017).

## 3 Tinder as a biological market: an evolutionary perspective

Given the features of Tinder and its users described in the previous section, we believe there are four main reasons that justify the application of an evolutionary perspective to the study of moral perception within Tinder users. First, from a functional point of view, assessing the morality of a potential dating partner seems to be highly relevant in an ambiguous social context such as Tinder. The app functions as a virtual intermediary that enables “face-to-face” encounters with strangers based on rapid evaluations of mostly visual (and sometimes a little textual) material. The fact that one knows little about whom one is dating, together with the tendency of offline post-match encounters to happen in an intimate context that may have risky consequences, could trigger the functioning of an evolved psychological mechanism that is sensitive to “short-term” ambiguous social interactions and potential social danger (Sevi, 2018; Sevi et al., 2018).

Second, empirical and anecdotal evidence suggest that the simple fact of being on Tinder may have some immoral connotations, which makes it essential to study the moral psychology of Tinder use (compared to other social media). For example, there is evidence that Tinder can be used for infidelity (Weiser et al., 2017), a behavior that is considered immoral within many moral codes (Graham et al., 2013). We believe that these facts make it especially relevant to study whether Tinder users are judging others according to moral character, and seeking to influence the judgments that others make of them. It might be the case that some users, especially women, tend to advertise through public or private textual messages on the system that they are not interested primarily in promiscuous sexual interactions, in effect protesting their innocence of the sexual “offences” of which many Tinder users are popularly suspected (see H2 and H4 for a discussion on gender differences in Tinder behavior).

Third, although the importance of attractiveness judgments in Tinder decision-making is widely assumed, some authors have claimed that sexual attractiveness may explain several aspects of moral psychology. Specifically, it has been claimed that the perception of desirable moral traits may be sexually attractive because these traits evolved to advertise mental fitness (Miller, 2007), suggesting that judgments of moral character may increase judgments of attractiveness in Tinder users.

Finally, we believe that previous theoretical and empirical research on social networks encourages the application of an evolutionary framework to the study of emerging technologies, especially when they rely on fundamental motives humans have such as mating and social relationships (Piazza and Ingram, 2015; Sevi et al., 2018). In particular, we believe that Tinder resembles a “biological market” (Hammerstein and Noë, 2016) in a very pure form, since there is a huge selection of partners to choose from, and very little cost involved in switching from a less attractive to a more attractive partner. As set out by Barclay (2013), biological market theory differs from earlier models of “partner choice” in evolutionary biology in three main ways. Firstly, competition in a biological market is based on relative rather than absolute value. Therefore, a Tinder user need not demonstrate that they are a paragon of morality, but simply that they are not as “predatory” as many of the other users on the site (Weiser et al., 2017). Secondly, as in economic markets, “buyers” in a biological market assess partners in an integrated manner based on a multitude of different traits. This means that “sellers” can make up for a lack of moral attractiveness by being attractive in other ways, a fact that we use to formulate our H4. Finally, market value tends to change over an individual's lifespan, and curves of change differ between the sexes, with females tending to lose value (due to declining physical attractiveness) in early middle age, when males may still be gaining value due to enhanced prestige (Buss, 1994).

As a result, we argue that, in addition to judgments of attractiveness, moral character is a good candidate for an evaluative dimension underlying rapid judgments of social desirability, especially for mate selection, and therefore a key part of the swiping and post-match decision processes. We are not saying, however, that moral character is more relevant than attractiveness in the Tinder context. Our claim is that, along with attractiveness, moral character plays a crucial role in swiping decisions, and an even more crucial role in post-match interactions. Future empirical research should address the relative importance of each dimension in swiping and post-swiping decisions. In the rest of this article, we build hypotheses to guide such research based on two related lines of investigation showing that (a) people draw multiple social inferences from minimal facial cues about a person, and (b) judgments of moral character are at the core of person perception.

## 4 H1: Social judgments extracted from faces should “colour” how non-face information about a person is processed

The Tinder interface heavily emphasizes photos and rapid judgments based on limited cues (mainly related to physical attractiveness) to make swiping decisions (Ranzini and Lutz, 2017). Typically, Tinder users try to display the most attractive, but still authentic, version of themselves in their profile pictures (Ward, 2017), using several tricks (e.g., camera angle, photo filters) to embody how they want to be perceived (Sedgewick et al., 2017). Therefore, whether a person is perceived as desirable or not desirable in Tinder largely depends on facial and bodily displays. Although we acknowledge that there is a large body of research on trait inferences from faces in social psychology (for a review, see Olivola and Todorov, 2017), in this section we only focus on studies on face perception and moral inferences because we believe that this research applies best to H1.

Although it is widely assumed that first impressions matter, the study of how these evaluations work in the age of social networking software, and what dimensions of evaluation are most important, requires further attention, especially if we consider how easily first impressions are formed and how inaccurate they can be. The state of the art suggests that the formation of first impressions is an automatic, extremely rapid process based on whatever evaluative information is available (Bar et al., 2006; Cone et al., 2017). Research on thin slicing (i.e., the ability of people to extract information about individual traits of others based on narrow windows of experience; Lykourentzou et al., 2017), has shown that when exposed to brief patterns of behavioral expressions, observers are even capable of making quite accurate judgments about a wide range of individual characteristics, such as socioeconomic status (Kraus and Keltner, 2009), scientific achievements (Kaczmarek et al., 2018), likelihood of being an appropriate teammate (Lykourentzou et al., 2017), among others.

### 4.1 Moral character and first impressions

On what evaluative dimensions are first impressions formed? Studies on how people gain first impressions of social targets have traditionally identified warmth (i.e., a social dimension that reflect traits related to perceived intent) and competence (i.e., a cognitive dimension that capture traits that are related to perceived ability) as two basic dimensions driving impression formation (Fiske et al., 2007). Being able to evaluate others’ positive or negative intentions and capabilities is crucial to survival in all human social environments, and thus these are crucial dimensions from an evolutionary point of view (Oosterhof and Todorov, 2008). Still, other authors have proposed a three-dimensional model of first impressions in which youthfulness-attractiveness constitutes a third dimension, related to cues of sexual selection (Sutherland et al., 2013).

In addition to these dimensions, over the last few years moral character has been proposed as a separate source of information that plays a crucial role in driving overall impressions of social targets (Goodwin et al., 2014; Goodwin, 2015). Although moral character information was previously considered as a sub-component of warmth (Cuddy et al., 2008), more recent research has noted that while the warmth dimension includes some traits that can be considered as highly moral (kindness, generosity, lovingness, etc.), other warmth traits seem much more distantly related to morality (extroversion, sense of humor, easygoingness, and so on; Goodwin et al., 2014). Some authors claim that evaluations of moral character can be better understood in the context of a person-centered approach to moral judgments, which focuses on persons rather than actions as the unit of analysis for moral judgments (Uhlmann et al., 2015). We think that in the case of social networks, a person-centered approach would suggest that users tend to give a “like” to the person, not just the post.

Supporting this view, it has been found that moral character information may be more important in impression formation than is warmth information, and that moral character may be the most important dependent variable in person perception research (Goodwin et al., 2014). According to the authors, this finding is congruent with both social functionalist and symbolic/existential considerations. With regard to the former, the facts that the morality of another person's character determines whether they are likely to be harmful or helpful to evaluator, and that these evaluations are crucial to assess the quality of potential social interactions (during the “post-match phase” of Tinder), support the necessity of a hyper-sensitive mechanism of moral inferences (see H2 for a discussion on this topic). In this vein, recent research showed that warmth and trustworthiness (two traits that are related with moral character; Goodwin et al., 2014) have a crucial role in long-term partnerships, but also play a role at early stages of mate selection such as in a speed-dating setting (Valentine et al., 2019).

Furthermore, it has been proposed that moral character plays a fundamental part in what it means to be human, and that moral traits are the most essential part of identity, the self and the soul (Strohminger and Nichols, 2014). Therefore, the importance of moral character in person perception may also reflect more symbolic considerations related with the “essence” of the person considered^2^. For instance, moral behaviors (Uhlmann et al., 2015), affective displays (Szczurek et al., 2012), attitudes (Bocian et al., 2018) and the perception of uncanny faces (Olivera-La Rosa, 2018) have been suggested to reveal moral character. Indeed, morality is so central to person perception that it makes sense to postulate an automatic pathway of moral inference. Research suggests that the perception of facial expressions showing deviant affective displays (i.e., lack of a startle reflex) may activate moral inferences (Szczurek et al., 2012; Tinwell et al., 2013), which is congruent with an evolutionary account of face evaluation. The need to rapidly infer another person's harmful intentions, in particular, justifies a fast and automatic but not necessarily completely accurate^3^ mechanism of moral judgments from faces (Oosterhof and Todorov, 2008).

### 4.2 “Face-ism” and first impressions

The face is a central source of social information. Research on “face-ism” (i.e., the tendency to stereotype people based on their facial appearance; Olivola and Todorov, 2017) has shown that people draw multiple social inferences from minimal facial cues about a person. As a result, a distinctive feature of social judgments based on facial appearance is that these judgments occur very rapidly and sometimes extend to preconscious stages of perception (Stewart et al., 2012). For instance, studies on trustworthiness judgments from unfamiliar faces found that these judgments are made after as little as 33–100 milliseconds (Willis and Todorov, 2006; Todorov et al., 2009). Bar, Neta, and Linz (2006) documented a similar processing threshold for threat judgments (but not intelligence judgments) made on unfamiliar faces. Indeed, the fact that intelligence judgments were less consistent at this processing times suggest that, when social traits are somewhat related with survival, those traits may be inferred from faces more quickly. Supporting these findings, there is evidence that untrustworthy-looking faces evoke a stronger response from the amygdala than trustworthy-looking faces, and that the more untrustworthy the face, the stronger the amygdala's response to the face, which supports the claim that unfamiliar faces are automatically evaluated on trustworthiness (Engell et al., 2007).

A crucial feature of personality inferences extracted from facial appearance is that these judgments are especially sensitive to attractiveness. The formation of attractiveness impressions from faces occurs regardless of one's intentions and they are difficult to inhibit once formed (Ritchie et al., 2017). This fact is especially relevant in Tinder decision making, given that Tinder users intend to selectively display attractive profile pictures in order to increase their chances of mating in the “biological market”, which ultimately allows that “average” Tinder users may appear much more attractive in their Tinder profile than they do in reality. Although the discussion of the mechanism of facial preferences exceeds the scope of this review, the state of the art suggests that attractiveness evaluation might reflect a social-evolutionary adaptation (Bzdok et al., 2010). Supporting this view, evidence from a meta-analysis showed that preference for facial beauty emerges early in development and is built on judgments of averageness, symmetry and sexual dimorphism (Rhodes, 2006). Further, Langlois et al. (2000) conducted 11 meta-analyses showing that there is strong agreement both within and between cultures about who is and who is not attractive. Crucially, they found that attractiveness may functions as an implicit marker of prosocial traits: attractive people are perceived to possess more positive behaviors and traits than unattractive people (e.g., better social skills, for an alternative explanation see Maestripieri et al., 2017). This Beautiful-is-Good stereotype is pervasive in social cognition and has been shown to bias social judgments in several domains (Eagly et al., 1991).

Of special relevance to this review is the finding that physical attractiveness influences moral inferences, specifically, by increasing the perception of socially desirable personalities and higher moral standards (e.g., “attractive people are friendlier than unattractive people”, Dion et al., 1972; Eagly et al., 1991). Interestingly, some research on the direction of attractiveness stereotyping suggests that most often, unattractiveness is a disadvantage more than attractiveness is an advantage in various domains of social judgment (e.g., altruism, intelligence; Griffin and Langlois, 2006). Further, the ubiquitous exercise of social inferences from physical attractiveness finds support in neuroscientific research which shows that the valuation of moral and aesthetic attributes relies on partially overlapping neural and cognitive mechanisms (e.g., medial orbitofrontal cortex and insular cortex, Tsukiura and Cabeza, 2011; Zaidel and Nadal, 2011), which some authors interpret as indicating that physical and personal attributes are coded along a single dimension by a shared evaluative brain circuit (Ferrari et al., 2017; for a detailed discussion on the relation between attractiveness and moral traits, see Miller, 2007).

Still, face-based social attributions may go beyond perceptions of physical attractiveness. Although several studies on the relationship between facial attractiveness and trustworthiness suggest that both evaluative dimensions may be closely interlinked (Bzdok et al., 2010), and that attractive people are trusted more than unattractive people (Palmer and Peterson, 2016), some studies suggest that facial typicality, rather than facial attractiveness, is the core factor predicting trustworthiness judgments (Said et al., 2010; Sofer et al., 2015; for a review, see Todorov et al., 2013). This effect may depend on the particular cultural context: a cross-cultural study found that different cultures (e.g., Japanese and Israeli) employed typicality cues when judging trustworthiness, and that own-cultural typical faces were perceived as more trustworthy than other-culture typical face (Sofer et al., 2017).

Critically, initial impressions may bias the acquisition of subsequent information by “coloring” subsequent evaluations (Cone et al., 2017). Some evidence based on self-report ratings suggests that the specific images we see of a person during an initial period of learning about their identity have an impact on subsequent judgments of attractiveness of that person, and that this mechanism may extend to other domains of judgment, such as trustworthiness (Ritchie et al., 2017). In the context of Tinder, this suggests that if a profile picture is evaluated as sufficiently positive or negative, it may bias the evaluation of the profile description (i.e., “about me” biographical taglines) or may directly halt the acquisition of any further information about a potential date. As a result, we predict that information depicted in the profile description will only be relevant (persuasive) for swiping decisions when first impressions are weak. Based on Ritchie et al. (2017), we can also speculate that those Tinder users who display images of themselves that are high in attractiveness or trustworthiness may be judged as more attractive or trustworthy, respectively, in the “post-match phase”). Further research is needed to test these predictions. For instance, we suggest that a cross-cultural approach may prove insightful in exploring these hypotheses, specifically, by examining whether Tinder users of different cultures differ in their reliance on pictorial information (vs. verbal information) when making swiping decisions. Interestingly, a recent study on Tinder profiles collected from Colombia and from the US found that, across both countries, women (relative to men) were more likely to use visual means in order to try to attract men to right-swipe; while men were more likely than women to include a verbal profile description, and to include information about their college major (Ingram et al., 2019).

## 5 H2: Moral character evaluations should be more likely to drive swiping decisions in female heterosexual users than in male heterosexual users

As the song says, “People are strange when you're a stranger, faces look ugly when you're alone”. Jim Morrison got it right: interacting with novel people may be threatening, or “creepy”. Indeed, it is surprising that despite the everyday popularity of the word “creepy”, psychological research on this emotional response is just beginning. There is agreement that creepiness is an unpleasant emotional response that arises from some ambiguity in a potential threat. Consistent with this view, McAndrew and Koehnke (2016) found that males (who are more physically threatening than females) were more likely to be perceived as creepy by both males and females, and that females were more likely to associate sexual threat with creepiness.

Watt et al. (2017) extended these findings by showing that creepiness largely resided in the eyes, that perceptions of creepiness were associated with violation of social norms, and that creepiness correlated positively with untrustworthiness. Based on their results the authors suggested, “It may be that ‘creepiness’ is more an emotionally based versus physically based judgment; reliant on emotional information gathered from certain key facial features of an individual” (p. 63). Therefore, the possibility that creepiness is an adaptive response directed to increase vigilance during periods of social uncertainty (e.g., interactions with novel targets) has been proposed by some authors (McAndrew and Koehnke, 2016). This claim fits well with an evolutionary account of unfamiliar social interactions: from an evolutionary perspective, it is crucial to detect diagnostic signals that reveal whether an unfamiliar target is an enemy or a friend (Becker et al., 2011). As a result, it is suggested that, when dealing with ambiguous situations, social perception operates according to the “smoke-detector principle”: psychological mechanisms err on the side of caution to minimize false-positive errors, at the expense of increasing false-negative errors (Nesse, 2005). Interestingly, the link between ambiguity and social danger is supported by neuroimaging research, which has shown that greater activation in the amygdala in response to ambiguous stimuli can be related to social anxiety (Griffin and Langlois, 2006; Thomas et al., 2001).

While separate from the literature on creepiness, insights from evolutionary theory favor the existence of sex differences in judgments of novel dating partners. According to parental investment theory (Trivers, 1972), females have historically needed to invest more time and effort in taking care of offspring than males. In this vein, the fact that women have much greater obligatory parental investment than men (due to pregnancy and breastfeeding), and, as a result, have potentially more to lose from a short-term, “casual” sexual encounter, ultimately leads to the evolutionary hypothesis that women tend to be more conservative and less risky in their mating choices. On the other hand, men are hypothesized to be more psychologically oriented towards short-term sexual relationships, prefer greater number of sexual partners over time, and require less time before consenting to sex (Buss and Schmitt, 1993; for a detailed discussion on this topic, see Gangestad and Simpson, 2000).

There is evidence that evolutionary mating theories have explanatory validity in Tinder research, and more widely in online environments (Piazza and Bering, 2009; Piazza and Ingram, 2015). As mentioned before, much evidence suggests that men are more likely to use Tinder for casual sex than women (Ranzini and Lutz, 2017; Sumter et al., 2016). Indeed, previous research on the mechanisms underlying the casual sex motivation of Tinder users suggests that sexual disgust sensitivity may function as an adaptive mechanism directed at protecting users from risky encounters. For instance, Sevi et al. (2018) found that men Tinder users with higher sexual disgust sensitivity and higher sociosexuality reported lower and higher motivation for casual sex in their Tinder usage, respectively. Interestingly, sociosexuality mediates the relationship between disgust sensitivity and the motivation to use Tinder for casual sex for women Tinder users, which is congruent with parental investment theory: regardless of their sexual disgust sensitivity, men could be more oriented towards casual sex than women, while only women who have low sexual disgust sensitivity may be motivated to use Tinder for casual sex (Sevi, 2018; Sevi et al., 2018).

As a result, judgments of moral character might be more important for women than for men because women have more to lose (in terms of time and effort) in the case of being pregnant from a casual sexual encounter where the man does not stay around to support his new family. Based on the reviewed literature, we believe that the fact that Tinder facilitates first contact with novel dating partners makes it likely that an automatic (and evolutionarily “conservative”) mechanism of social inferences may be involved in swiping decisions. Crucially, this literature suggests that in a context of social uncertainty (such as is the case in Tinder-based interactions), rapid assessment of potential social danger (e.g., violence, deceit, rape) inferred from facial features may drive decision-making. As previously discussed, the fact that judgments of moral character are crucial to assess the quality of potential social interactions, combined with males being more physically threatening than females and with females having more to lose from casual sexual relationships, makes us hypothesize that this mechanism may play a larger role in female heterosexual users than in male heterosexual users (H2).

We can also speculate (as a derived hypothesis) that judgments of moral character assessing potential violent behavior would be more important for heterosexual than for homosexual users, given that sex differences in physical threat and the potential risk of pregnancy would be less relevant issues in the latter context. There are some previous studies on homosexual Tinder users. For instance, a study with bisexual women showed that they found Tinder safer than offline interactions with strangers, but at the same time, they felt that Tinder created new risks such as online deception or “catfishing” (Pond and Farvid, 2017). Another study with gay men in London found that gay users within this community displayed a less sexualized digital identity in Tinder, when compared to other gay dating apps. The authors concluded that even a highly stereotyped dating app like Tinder may be reinterpreted by gay men in particular contexts (MacKee, 2016). Further research could explore these hypotheses, for instance, by assessing whether the influence of attractiveness and moral character on swiping decisions varies between users of different genders and with different sexual orientations.

## 6 H3: Profile pictures that apply “unnatural” photo filters may facilitate social avoidance rather than social desirability

The facts that Tinder users typically make rapid judgments of other users based on their profile pictures, and that many of these pictures apply photo filters, make it relevant to discuss the potential role of photo filters in social perception. Although we are aware that the perception of photo filters can cause a variety of emotional and/or social evaluations (e.g., amazement, disappointment, impression of “faking”), in this section we focus on the potential moral effects of applying “unnatural” photo filters in a context of social uncertainty such as Tinder, because it relates to the hypothesized role of moral character judgments in Tinder swiping decisions.

Research on the “uncanny valley” hypothesis (Mori, 1970/2005) offers some insights into the perception of “odd” faces and their social implications. Briefly, this hypothesis posits that entities which look quite close to being human, but not completely human, can produce negative feelings in an observer: the more human-like an entity looks, the more pleasantly it is experienced, until a point is reached at which it starts to elicit an unpleasant emotional response: the uncanny feeling (UF). The realm of the uncanny seems to be broad. Entities such as androids, sex toys, wax figures, dolls, CGI characters, cartoons, mannequins, clowns, masked or facially scarred individuals, or even Botox users have been previously associated with the uncanny response (Pollick, 2010; Smith, 2014).

Some researchers have argued that the UF is caused by an inconsistency between the human-likeness levels of specific cues (MacDorman and Chattopadhyay, 2016; Seyama and Nagayama, 2007; for comprehensive reviews of the most influential psychological explanations of the UF, see Kätsyri et al., 2015; Wang et al., 2015). Indeed, substantial evidence indicates that the human visual system has acquired a heightened sensitivity in discriminating facial features (Hassin and Trope, 2000; Nesse, 2005; Simpson et al., 2011). This perceptual process appears to be highly automatic, facilitating that any incongruent or “odd” facial feature activates our hyper-sensitive perceptual alarm system (signaling that “something may be wrong”). For instance, there is evidence that perceiving small deviations from human appearance produces large prediction errors in brain regions associated with the recognition of human faces (Chattopadhyay and MacDorman, 2016). As a result, it is plausible that perceptual mismatches triggered by any atypical facial feature (e.g., photo filters depicting grossly enlarged eyes) may violate our a priori “natural” expectations, causing an observer to experience the UF.

In addition, some research on the UF suggest that this emotional response may bias how uncanny targets are perceived morally. In this vein, there is evidence that perceptions of psychopathy may be involved in the UF. Tinwell, Nabi, and Charlton (2013) showed that aberrant facial expressions (e.g., inadequate upper facial animation in virtual characters) led to a perception of psychopathic traits, which ultimately triggered the UF. According to the authors, this finding suggests that the UF may function as an avoidance response towards those targets evaluated as emotionally unpredictable (i.e., signaling that a person's intentions are unpredictable and potentially dangerous): “For survival purposes, the human default interpretation in such circumstances has possibly evolved to be one of ‘erring on the side of caution’ and preparedness for the possibility that we are in the presence of a being with psychopathic-like traits and thus potential danger” (Tinwell et al., 2013, p. 1623). Accordingly, it has been suggested that the UF functions as an emotional signal that something is “not right” with the perceived moral character of a target, and that said target therefore needs to be avoided (Olivera-La Rosa, 2018). Recently, these hypotheses were tested using an implicit associations paradigm: across five Single-Target Implicit Association Tests the authors found support only for a slight association of the UF with moral disgust (relative to fear), but not evidence of an implicit link between the UF and cognitions of psychopathy; Villacampa et al. (2019).

**Figure 1:**
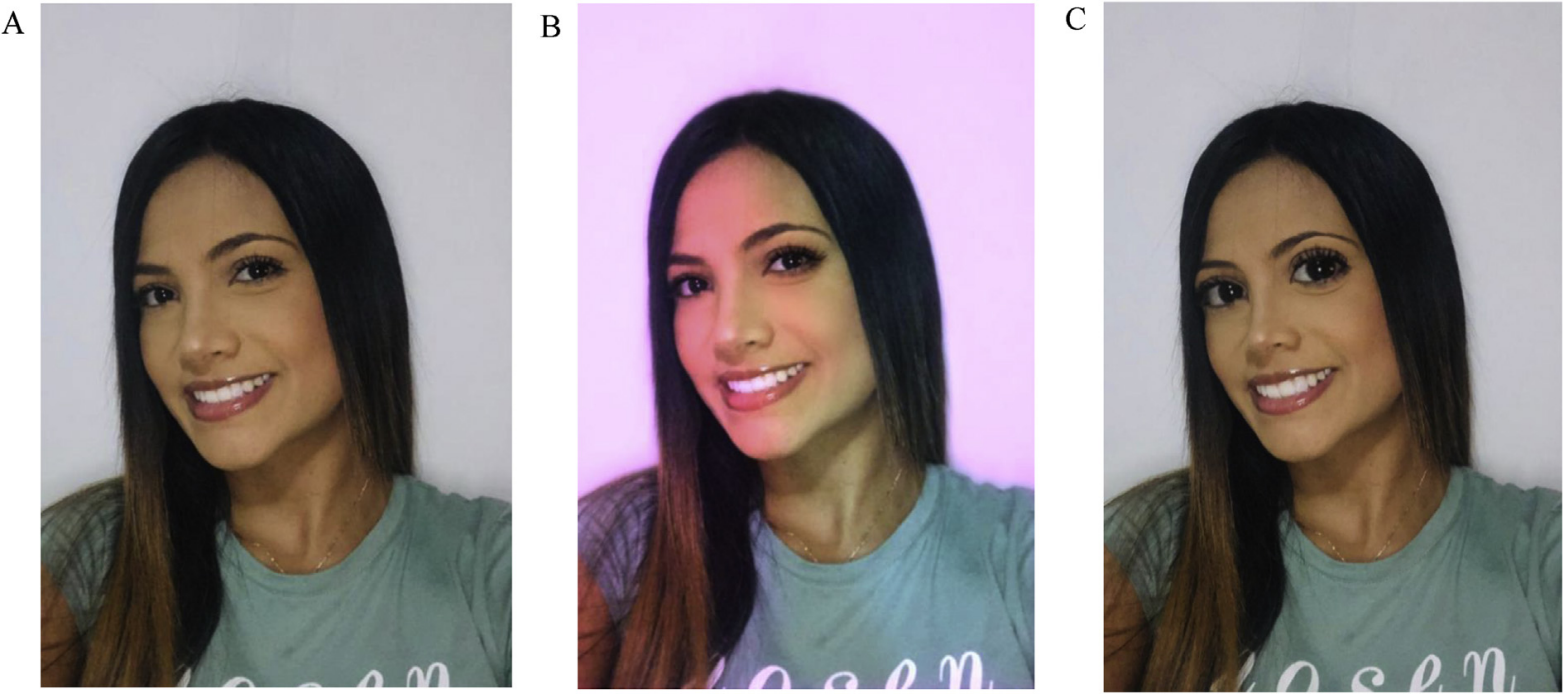
Examples of photo filters. From left to right, the presented images illustrate profile pictures that apply no filter (1A), slight photo filter (IB), and “unnatural” photo filter (IC). The portraits are modeled by a research assistant.

Based on this literature, we hypothesize that Tinder profile pictures that apply “unnatural” photo filters (e.g., extremely enlarged eyes) may facilitate social avoidance rather than social desirability at early stages of social interactions (H3). To the best of our knowledge, no study has assessed the uncanny valley in the context of Tinder behavior, which may be a fruitful research opportunity given the proliferation of photo filters in profile pictures. From this point of view, it should be noted that the UF may be stronger in the initial stages of social interaction (i.e., first impressions). Złotowski et al. (2015) found that the UF drops after repeated interactions with an android, which indicates that learning that a target is not harmful diminishes the automatic negative emotional response to the novel stimulus. Given the importance of the reviewed literature in the context of Tinder interactions, we propose an experimental approach in which participants both explicitly and implicitly evaluate the uncanniness and social desirability of a large sample of real-world Tinder profiles (for a similar approach to the uncanny valley see Mathur and Reichling, 2016; Mathur et al., 2019). It should also be noted that modified versions of this hypothesis could apply to other social networks in which filters are widely used, such as Snapchat and Instagram. Finally, we would like to make it clear that we do not claim that “any” photo filter may trigger the UF in Tinder users. Our argument has been, rather, that when used in a way that denaturalize the human face, photo filters can activate our perceptual alarm system, causing the UF and perceptions of deviant morality (Figure 1). Future research should test this assumption.

## 7 H4: Less attractive mates need to work harder in the post-match phase to demonstrate their moral character

As suggested in Section 2, Tinder may be a remarkably pure example of a biological market, in which the market participants have access to a huge range of potential partners to choose from, and in which there are rarely negative consequences of swapping out of a bad choice. This is largely because, after swiping right and finding that they have a match, Tinder users have an additional opportunity to evaluate moral character (as well as other factors such as warmth and competence) in the chat that must be initiated if they are to have any sexual contact with a match. We refer to this chat as the “post-match phase” of Tinder use.

Although, as discussed above, moral character may be implicitly evaluated in the earlier swiping phase, for example by viewing strange filters unfavorably, it is likely to be evaluated much more systematically and explicitly in chat-based interactions, for example by asking someone if they are married or if they can provide social media links (for corroborating information). In Tinder there thus seems to be quite a clear separation between two aspects of biological markets as described by Barclay (2013; see also Kummer, 1978; Tooby and Cosmides, 1996). These are the evaluation of a potential partner's *qualities* (their ability to provide benefits, which in the case of a mating market boils down to attractiveness) and of their *tendencies* (their willingness to provide benefits).^4^ Tendencies are the more complex aspect of this formula, since they can encompass many different aspects: for example, a heterosexual man may be interested in a woman's willingness to engage in casual sex, whereas a heterosexual woman may take that for granted and instead be more interested in a man's lack of commitments to other women.

Most tendencies, moreover, can be thought of as reflecting a person's essential moral character in some way (Bloom, 2010), as opposed to qualities, which reflect more material factors such as physical attractiveness and economic resources. An important tenet of biological markets theory is that qualities and tendencies are summative: therefore, someone who is low in mating quality can still achieve mating success if they score highly on relevant tendencies, and vice versa (Barclay, 2013). What this means for Tinder-based interactions is that someone who is lower in quality as a potential mate will have to work harder in the post-match phase in order to demonstrate both their willingness to pair up and their moral character; for instance by initiating the chat, writing more, flirting, questioning the interaction partner about their life, volunteering details about their own life, responding to questions if asked, and demonstrating general good humor and cooperative tendencies.

Furthermore, what dictates “quality” in potential mates is highly dependent on sex: in heterosexual markets, women are generally of more value than men, and physical attractiveness is more important in evaluating women than men, whereas access to material resources is more important in evaluating men (Buss and Schmitt, 1993). The same authors point to differences in age of mate preferences between the sexes: this was backed up by a study of hundreds of singles ads which found that women consistently looked for men of their own age or up to a few years older, whereas men progressively preferred women of a greater age difference below them as they grew older (Kenrick et al., 1995). These factors lead to clear predictions, both within and between sexes, about who will have to invest more in verbal behaviors aimed at demonstrating willingness and moral character during the post-match phase. In general, we expect less attractive partners to have to do more of this sort of work than more attractive partners, men to have to do more than women, older women to do more than younger women, and men with less resources to do more than men with more resources.

## 8 Conclusions & limitations

In summary, the evidence reviewed in this article indicates that moral character may be an evaluative dimension underlying swiping decisions, influencing rapid judgments of mate selection in Tinder. We argue that Tinder users not only make judgments based on physical attractiveness, but also based on perceptions of moral character. By addressing the most striking findings on moral character and face perception, we conclude that first impressions extracted from faces are strongly linked not only with simple judgments of attractiveness, but also with judgments of moral character and social desirability. This perhaps largely automatic cognitive pathway appears to be especially salient in the case of unfamiliar targets, which is typically the case with Tinder users.

This article is intended as a catalyst for further research. Therefore, we aim to call the attention of scholars to the value of research on social perception in the context of behavior on Tinder (and similar online dating services that are based primarily on visual evaluation). Specifically, these observations not only help to locate the study of Tinder from the standpoint of robust lines of research (e.g., biological markets, face perception, first impressions, thin slicing), but also to incorporate exciting new developments in the study of creepiness, the uncanny valley and their potential moral connotations. With this aim, we have presented testable hypotheses motivated directly by the revised literature. We do not, however, claim to have exhausted all domains of social perception. As we have mentioned, there are many studies on face perception, attractiveness and social judgments (for reviews see Langlois et al., 2000; Maestripieri et al., 2017; Olivola and Todorov, 2017), and a comprehensive review of that literature largely exceeds the purpose of this work, which is to provide provocative insights for future research on online dating systems like Tinder and other visually based social networks such as Instagram and Snapchat.

In this vein, we emphasize that this article has focused on face perception, which is a key feature of the Tinder user interface. Nevertheless, we acknowledge that Tinder users commonly depict other type of pictures (and videos) in their profiles, such as pictures of their own bodies (which are sometimes highly sexualized). Beyond face displays, we expect that future research should address the psychological mechanisms involved in the perception of Tinder profiles depicting sexualized displays, given the importance of social perception in the age of Tinder.

## Declarations

### Author contribution statement

All authors listed have significantly contributed to the development and the writing of this article.

### Funding statement

This research did not receive any specific grant from funding agencies in the public, commercial, or not-for-profit sectors.

### Competing interest statement

The authors declare no conflict of interest.

### Additional information

No additional information is available for this paper.

## Footnotes

1 Recently, users are allowed to upload extremely brief (up to 2 s) videos to their Tinder profile.

2 As suggested by Bloom (2010; xii preface): “One of the most interesting ideas in the cognitive sciences, which is that people naturally assume that things in the world-including other people-have invisible essences that make them what they are. Experimental psychologists have argued that this essentialist perspective underlies our understanding of the physical and social worlds, and developmental and cross-cultural psychologist have proposed that it is instinctive and universal. We are natural-born essentialists.”

3 It may be adaptive to have a “negativity bias” towards the possibility of harmful intentions (cf. Baumeister et al., 2001), which would reduce accuracy by causing some individuals to be characterized as less moral than they actually are.

4 A third aspect, *availability*, is in most cases taken care of by the fact that someone is on Tinder and is geographically close to their match, since proximity is one of the main input variables to the algorithm that presents potential matches to Tinder users.
